# Abnormal Elongations of HOX Gene Clusters May Cause Cancer

**DOI:** 10.3389/fcell.2018.00025

**Published:** 2018-03-12

**Authors:** Spyros Papageorgiou

**Affiliations:** Institute of Biosciences and Applications, National Center for Scientific Research “Demokritos”, Athens, Greece

**Keywords:** Hox cluster elongation, Hox clusters as elastic springs, Hox genes and cancer, Hox gene mutations, Hox gene collinearity

## Background

Hox gene activation is crucial for normal development of most organisms. Because of its importance, in both development and disease, it is intensively studied. Up to now, molecular methods have been almost exclusively used in order to explore the underlying mechanisms in normal embryonic growth (Tarchini and Duboule, 2006; Tschopp et al., 2009). The clustering of Hox genes is of particular interest in the case of the vertebrates (Duboule, 2007). Using classical genetic methods, Lewis discovered the fundamental property of Hox gene collinearity (Lewis, 1978). According to this collinearity, the ordered Hox1, Hox2, Hox3, etc., genes along the telomeric (3′) to the centromeric (5′) direction on the chromosome are activated in the same order in the ontogenetic units along the Anterior (head)—Posterior (tail) A/P axis of the embryo.

The main feature of Hox gene collinearity is its multiscalar nature: the compact size of an inactivated Hox cluster is about 150 nm whereas the linear size of an early (mouse) embryo is about 1 mm. The two sizes differ by more than 4 orders of magnitude therefore the molecular mechanisms alone are not adequate to describe all Hox cluster collinearity data. Physical laws are more suitable to interrelate phenomena and entities extending over so different spatial scales, as for example the electrons and the nucleus of an atom. In the simplest case of the hydrogen atom, the electron is located in an “electronic cloud” around the nucleus (a proton). The size of the “electron cloud” is more than 4 orders of magnitude greater compared to the size of the atomic nucleus. The Coulomb force keeps the electron on track around the nucleus while the long-range structure of this force covers the space in between.

The aforementioned multiscalar physical example motivated the formulation of an alternative model, the biophysical model (BM), to explain the collinear transcription in the Hox gene clusters (Papageorgiou, 2001, 2006). A simple heuristic pulling force F was introduced depending on two factors N and P giving rise to the following equation:

(1) $$\begin{array}{r} \text{F}=\text{N}\cdot \text{P} \end{array}$$

In Equation (1), F is a Coulomb-like force where the factor N stands for the “negative charge” in the microscale of the Hox gene cluster. The “positive charge” P factor reflects the macroscopic component of F. Along the A/P axis a morphogen gradient is established with the low and high morphogen values located at the head and tail of the embryo respectively. The morphogen is transduced inside every cell and positive molecules are produced, transported and fixed at a specific location opposite the telomeric end of the Hox cluster. Historically, the morphogens were fictitious until their existence was confirmed (Towers et al., 2012). It is similarly legitimate to assume the existence of P molecules since many other transduced molecules with specific properties have been observed in the cell nucleus like protein SMAD2 (Shimizu and Gurdon, 1999; Simeoni and Gurdon, 2007). The pulling force F extrudes sequentially the Hox genes Hox1, Hox2, Hox3,…toward the transcription factory domain where transcription is possible (Papageorgiou, 2017). The BM can consistently explain the existing genetic engineering data of gene deletions, duplications and transpositions (Papageorgiou, 2011; Gordon and Gordon, 2016).

## Hox cluster as an expanding elastic spring

**Figure 1:**
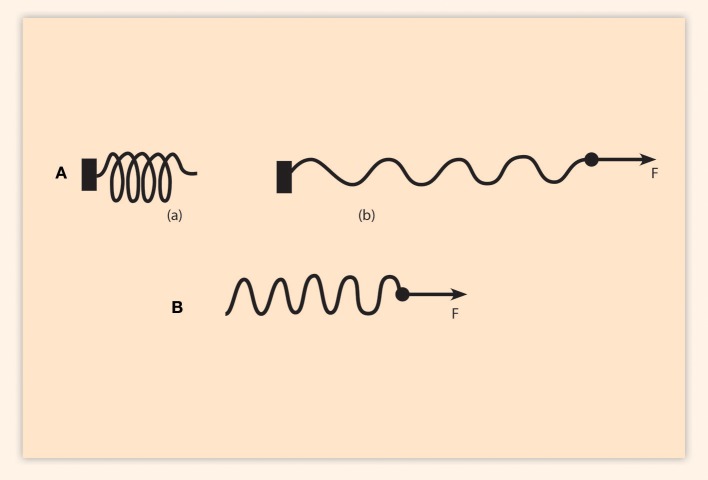
Adapted from Papageorgiou (2017). Elastic spring elongation. **(Aa)** An uncharged elastic spring with fixed left end and free right end. **(Ab)** Pulling force F elongates the spring in the direction of the force. **(B)** The fixed end of the spring is cut-off. The same force F causes: (1) a slide of the spring to the right. (2) An elongation of the spring. This elongation is smaller than the elongation of **(Ab)**.

According to the BM, the sequential pulling of the Hox genes looks like an expanding elastic spring (Papageorgiou, 2012). This expanding spring description is a simplification of the detailed local DNA interactions that sum up to an integral collective picture. The inactive compact Hox cluster is represented by an uncharged elastic spring whose one end is free to move and the other end is fastened (Figure 1Aa). When a force F is applied on the telomeric end of the cluster, the Hox1 is extruded toward the transcription factory domain. In the mechanistic analog, the spring expands as shown in Figure 1Ab. The above interpretation was applied to explain the findings of two important deletion experiments (Kondo and Duboule, 1999). In the first experiment (Exp1) the transcription of the probe gene Hoxd10 was analyzed. In the wild type mouse embryo, Hoxd10 starts being transcribed at stage E8. In Exp1 the posterior genes (Hoxd11, Hoxd12, Hoxd13) were deleted and no Hoxd10 expression is observed at stage E8. In Exp2, besides the deletion of the posterior (Hoxd11, Hoxd12, Hoxd13) genes, the neighboring centromeric region to the HoxD cluster is also deleted (Kondo and Duboule, 1999). At stage E8 the result of Exp2 is unexpected: the transcription of Hoxd10 is prematurely observed. This indicates that the deletion of the neighboring centromeric region affects strongly the probe Hox gene transcription.

The elastic spring representation of the BM can explain these findings (Papageorgiou, 2012). In Exp1 the posterior Hoxd deletions reduce the factor N in Equation (1), therefore F decreases. In order to extrude Hoxd10 the force F must recover its strength, hence P must increase. As a result, at the macroscale, posteriorization of Hoxd10 accompanied by a retardation of transcription occurs (Papageorgiou, 2011). Therefore, at stage E8 the transcription of Hoxd10 is not yet initiated. The multiscalar nature of collinearity is manifested via the interplay between factors N and P in Equation (1) (micro- and macro-dimensions respectively).

In Exp2, the premature transcription of Hoxd10 is attributed to the cutoff of the fastening region of the elastic spring (Figure 1). In this representation, the applied force F shifts the spring toward the telomeric side leading to a premature transcription of Hox10 associated with an intensity variation of transcription (Papageorgiou, 2012, 2017). The result of Exp2 (Kondo and Duboule, 1999) is interpreted by the BM as the drastic outcome due to the cutoff of the fastening domain of the spring. In this spirit, it is expected that milder mutations (spontaneous or experimental) contained in the neighboring centromeric domain can affect the fastening efficiency of the elastic spring so that the spring may become tighter or looser. For such milder mutations, a given pulling force will produce a transcription variation of the Hox genes compared to the wild type transcriptions (e.g., overexpression or premature Hox gene expression). It is legitimate therefore to consider that these milder mutations are causally connected to the Hox gene transcription modifications. In this interpretation the Hox gene expression variations are due to mutations of the centromeric domain of the Hox cluster. Gene Evx2 may be contained in this centromeric domain. It is documented in a recent review that conserved non-coding elements (CNEs) tend to cluster in the vicinity of genes with regulatory roles (Polychronopoulos et al., 2017). Disruption of these CNEs contributes to cancer. It would be interesting to explore if such CNEs are associated with the centromeric domains fastening the Hox clusters.

## A passage to abnormal hox gene expressions and cancer

It has been suggested for a long time that abnormal Hox gene expression may cause severe diseases. For instance, it was observed that dysregulation of Hox genes is related to acute myeloid leukemia (Alharbi et al., 2013). Or that Hox gene cluster expression is increased in myelodysplastic syndrome patients (Xu et al., 2016). In another work, overexpression of several Hox genes (including HoxA13 and HoxD13) were observed in cases of ovarian cancer (Kelly et al., 2016). In these studies the symptoms of malignancy are described as concurrent with the variation from normal Hox gene cluster expressions and they are not or could not be proved to be linked.

As a working hypothesis it is assumed that an abnormal Hox gene expression leads to a malignacy. From the evidence presented above, the DNA variation of the fastening domain of the Hox cluster affects the normal Hox cluster activation (Kondo and Duboule, 1999). Therefore a mutation inside this fastening centromeric domain may cause this malignancy. It could be expected that the physical-mechanical origin of these mutations might lead to distinct malignancies compared to the malignancies of molecular origin alone. The physical cause may convey some particular features that can be traced along the pathway to the final malignancy (e.g., degree of robustness). The BM in its elastic spring formulation leads to a cause and effect relation: the mutations contained in the fastening centromeric domain (denoted Dc) cause abnormal Hox gene expressions which subsequently lead to some forms of cancer. The above opinion-hypothesis can be indirectly tested by systematic digging in the comprehensive Data Bases of several organisms as indicated below. It is crucial to verify this hypothesis.

Exhaustive lists of cancer-driving mutations have been identified in specific cancers as, for instance in kidney cancer (Long et al., 2017) or lung cancer (Rauch et al., 2007). From the Data, a comprehensive list of lung cancer driving mutations in humans can be documented and the Dc domain identified. This centromeric domain Dc next to the Hox clusters is a candidate fastening domain of one of the Hox clusters (for simplicity, Hox stands for one of the 4 HoxA, HoxB, HoxC, and HoxD clusters). The size of Dc is not rigidly fixed and can flexibly vary. Furthermore, from the Human Genome Data, several other random domains Dr can be selected whose size is equal to Dc (Dr = Dc). The location of these Dr domains can be randomly distributed in the human genome. The malignancies caused by the mutations contained in a certain Dr can be compared to the malignancies due to the mutations contained in Dc. As argued above, the two sets of malignancies could be clearly distinguished (e.g., in robustness, persistency, time of appearance, etc.). For the mutations in Dc the following pathway is expected:

Mutations in Dc → Abnormal Hox Gene expressions → Cancer

Another application of extracting information from digging in the Data Bases is the case of acute myeloid leukemia (AML). In AML, overexpression of specific HOXA and HOXB genes is detected (Kontro et al., 2017). It would be interesting to explore whether AML can be related to mutations contained in Dc. If true, this would be important for two reasons: firstly as a further verification of the BM and secondly because it would indicate the cause of a certain kind of cancers.

## Conclusion

New technological advances and novel methods (like superresolution imaging-STORM) enabled the determination of the physical-geometric transformations of the Hox clusters during Hox transcription. For example, the physical elongations of the HoxD cluster were analyzed at the different stages of gene activation (Fabre et al., 2015). It was observed, in particular, that this geometric restructuring of the DNA fiber predates transcription. These findings verify some of the BM predictions (Papageorgiou, 2017). According to the current trend, Hox gene collinearity is treated superficially as a mere property of Hox gene clusters. The conventional interpretation of the data is based on biomolecular mechanisms alone ignoring the physical involvement. This “current dogma” on collinearity was recently challenged by Durston and Zhu. These authors emphasize the importance of multiscalar phenomena as combined with a spirit of “going back to basics” (Durston and Zhu, 2017). This is exactly what inspired the biophysical model several years ago (Papageorgiou, 2001).

The integration of the physical and biomolecular contributions in explaining the Hox gene cluster activation proved already fruitful in normal development (Papageorgiou, 2017). The exploration of the origin of some forms of cancer is evidently of pivotal importance for both Biology and Medicine. As stressed already, it is worth trying to verify or reject the predictions described above. The plethora of Data accumulated in Data Bases (e.g., the Cambridge Big Data) enable the realization of this quest.

## Author contributions

The author confirms being the sole contributor of this work and approved it for publication.

### Conflict of interest statement

The author declares that the research was conducted in the absence of any commercial or financial relationships that could be construed as a potential conflict of interest.
